# Role of Molecular Biology in Cancer Treatment: A Review Article

**Published:** 2017-11

**Authors:** Aman IMRAN, Hafiza Yasara QAMAR, Qurban ALI, Hafsa NAEEM, Mariam RIAZ, Saima AMIN, Naila KANWAL, Fawad ALI, Muhammad Farooq SABAR *, Idrees Ahmad NASIR

**Affiliations:** 1. Center of Excellence in Molecular Biology, University of the Punjab, Lahore, Pakistan; 2. Institute of Molecular Biology and Biotechnology, University of Lahore, Lahore, Pakistan; 3. Dept. of Plant Breeding and Genetics, University of Agriculture, Faisalabad, Pakistan; 4. Southern Cross Plant Science, Southern Cross University, Lismore, Australia; 5. Centre for Applied Molecular Biology, University of the Punjab, Lahore, Pakistan

**Keywords:** Cancer, Oncogenes, Proto-oncogenes, Mutagenesis, Viral infection, Tumor, CRISPR

## Abstract

**Background:**

Cancer is a genetic disease and mainly arises due to a number of reasons include activation of onco-genes, malfunction of tumor suppressor genes or mutagenesis due to external factors.

**Methods:**

This article was written from the data collected from PubMed, Nature, Science Direct, Springer and Elsevier groups of journals.

**Results:**

Oncogenes are deregulated form of normal proto-oncogenes required for cell division, differentiation and regulation. The conversion of proto-oncogene to oncogene is caused due to translocation, rearrangement of chromosomes or mutation in gene due to addition, deletion, duplication or viral infection. These oncogenes are targeted by drugs or RNAi system to prevent proliferation of cancerous cells. There have been developed different techniques of molecular biology used to diagnose and treat cancer, including retroviral therapy, silencing of oncogenes and mutations in tumor suppressor genes.

**Conclusion:**

Among all the techniques used, RNAi, zinc finger nucleases and CRISPR hold a brighter future towards creating a Cancer Free World.

## Introduction

Cancer is a genetic disease. The expression of oncogenesis is an important event in early stages of tumor formation. Oncogenes are activated through two mechanisms: either by infection of cells by tumor viruses or by mutation of cellular proto-oncogenes (which are usually normal) to oncogenes. Then tumors originate by oncogenic transformation of only a single cell. Some tumors adopt the ability to escape the site of their origin and intrude other parts of the body. This process is called metastasis. Solid tumors i.e. sarcomas, could be transferred from one animal to another using Rous sarcoma virus. Tumors could be caused either by the addition or by expression of genetic material, which in this case was viral DNA, to normal cells. Rous was presented a Nobel Prize for his work. In 1978, tumors of nonviral origin were also discovered (1).

## Methods

This article was written from the data collected from well-reputed `databases of PubMed, Science Direct, Springer and Elsevier groups of journals. The data were collected in the form of research and review articles on the use of molecular biology for cancer research, which was summarized to form an improved and advance review article. The following key words were used to search the databases: Cancer, Oncogenes, Proto-oncogenes, Mutagenesis, Viral infection, Tumor, CRISPR. Articles were verified published until 2015.

## Results and discussion

Further, the relation between carcinogens and mutagens saying was described that many carcinogens are also mutagens. Mutations in normal genes might cause them to become cancerous and this could happen due to any damage to DNA. This hypothesis got further support after molecular biology tools were applied in cancer research (2). Other than expression of oncogenes, cancer may also arise due to the loss or damage of tumor suppressor genes that check and control the cell growth. In many cases, both reasons contribute to causing cancer (3).

### Oncogenes in cancer development

Oncogenes are the tumor causing genes and have important role in development of many cancers. In 1970, *SRC* oncogene was discovered in chicken retrovirus. As a result of some mutation in the otherwise normal proto-oncogenes, their deregulation occurs and uncontrolled proliferation of cells starts and leads to cancer (4). At genomic level, only single oncogenic allele is required to alter normal gene function because of its dominant property. The origin of oncogene can be cellular i.e., from inside the body or viral i.e., from some virus (3).

Gene Duplication, addition, insertion, deletion or chromosomal translocation, chromosomal rearrangement of certain proto-oncogenes alters their function and converts them into oncogenes. These mutations overexpress the protein to an uncontrolled level, which may lead to tumor. These mutations may occur due to external factors or internal factors or both like viral infection, radiation or chemicals, injury and disease (4). Among these mutations, viral infection is the rare cause of oncogene activation in animals but is of great importance for understanding oncogene function.

### Viral infection

Retroviruses or DNA viruses cause viral infections. These viruses infect the host either by inserting oncogenes in host chromosome, interfering proto-oncogene transcription factors/regulators or by inserting homologous sequences corresponding to normal protooncogene of host. For example, retrovirus carrying *SRC* oncogenes infects the host, integrates viral chromosome in host chromosome, further divides the viral progeny and infects the surrounding cells, inducing overexpression of cellular normal genes and deregulated proliferation of cells in order to cause cancer (5).

### Types and classification of oncogenes:

Oncogenes can be classified into five classes based on protein products formed by mutation or deregulation of proto-oncogenes. These include growth factors, growth hormone/factor receptors (GRFs), serine/threonine kinases, GTPase molecules and, transcription factors. Mutations in the growth factors can lead to several types of cancers such as fibrosarcoma, glioblastoma (brain cancer), osteosarcoma (bone cancer) etc. (6, 7).

In several tumors, “ligand-binding domain” deletions of Epidermal Growth Factor Receptor cause successive activation of receptor even in absence of ligand by transmembrane protein carrying tyrosine-kinase activity. This activation causes interaction with further cytoplasmic proteins like “SRC domain” and leads to deregulation of numerous signaling pathways. Mostly in gastrointestinal, breast and lung cancers, GFR mutations occur (4). Similarly, overexpression of Raf-1 kinase and cyclin-dependent kinases due to uncontrolled phosphorylation may cause many cancers such as thyroid and ovarian cancer.

Deregulated activation of GTPases such as Ras, causes activation of MAPK pathway and uncontrolled signaling and division of cells cause several cancers such as myeloid leukemia. Transcription factor proteins are products of protooncogenes. The mutation, translocation or rearrangement of these causes overexpression of gene and unwanted consecutive transcription of target gene that leads to any types of cancers such as pancreatic and lung cancer (6).

### Role of oncogenes to treat cancer

The oncogenes are targeted to treat oncogenic cancer. Several oncogenes discussed above are targeted by drugs and gene therapies to inhibit, arrest, regulate or senescence their genes. For example, Imatinib (ABL kinase inhibitor) or Gleevec is used to treat BCR-ABL. Gefitinib or Iressa, erlotinib or Tarceva are used to target EGFR. VEGF oncogenes are targeted by bevacizumab or sorafenib. Sorafenib is also used to downregulate or inhibit B-Raf oncogene. These agents/drugs are used, sometimes in combination, for chemotherapy to inhibit proliferation of oncogenes or to downregulate signaling oncoproteins in several signaling pathways to treat oncogenic cancers (8). However, it is difficult to target “non-kinase oncogenes” through drugs such as Myc and Ras.

### Tumor suppressor genes in cancer

Tumor suppressors play their role by inhibiting cellular proliferation and tumor development. In most of the tumors, inactivation of the tumor suppressor genes eliminates the negative regulation of these genes over cellular proliferation that leads to abnormal cell proliferation, therefore, causing cancer. Tumor suppressor genes have “loss of function” mutations because they develop cancer by inactivating their inhibitory effect on cell proliferation. For a tumor suppressor gene to promote tumor development, both copies of the gene must be inactivated because one copy is sufficient for controlling cell proliferation. These mutations act recessively (9).

### Role of Tumor Suppressor Genes

The protein products tumor suppressing genes are found to play the following important functions:

- Enzymes involved in DNA repair
- Checkpoint-control proteins arresting the cell cycle in case DNA is impaired or chromosome abnormality.
- Proteins promoting programmed cell death (apoptosis)
- Inhibiting cellular growth and proliferation by acting as receptors for hormones.
- Intracellular proteins which regulate or inhibits movement through a specific stage of cell cycle (10).

### Role of Tumor Suppressor Genes in Cancer Wilms’ Tumor 1 Gene

Some tumor-suppressing genes act as transcriptional regulatory proteins. For example, the product of WT1 gene which is a repressor protein and acts by suppressing transcription of many growth factor-inducible genes. WT1 is made inactive in Wilms’ tumors (which is a tumor in kidney found in children). Insulin-like growth factor II is the target of WT1 gene, over-expressed in Wilms’ tumors, thereby contributing to abnormal cell proliferation (11).

### Retinoblastoma and INK4 Genes

Several tumor suppressor genes regulate cell cycle progression through a specific stage e.g. protein products of Rb and INK-4 genes. Retinoblastoma is the tumor of the eye. Two mutagenic events are required for the retinoblastoma development in sporadic cases whereas only one mutagenic event is needed in individuals with inherited form of the disease in which it displays autosomal dominant inheritance. In normal cells, Cdk2 and cyclin D complexes regulate the entry through the constraint point, thereby phosphorylating and inactivating pRb. pRb also impedes the entry through the constraint point in the G_1_ phase of the cell cycle by repressing the transcription of many genes involved in cell cycle advancement. The INK4 tumor suppressor gene also regulates movement through the restriction point by encoding Cdk inhibitor p16. Inactivation of INK4 results in uncontrolled phosphorylation of Rb (10).

### p53 Tumor Suppressor Gene

The p53 plays its role by regulating cell cycle and programmed cell death. The p53 can arrest the cell cycle upon DNA damage. It allows the DNA to repair or cause the programmed cell death (apoptosis). This is achieved by activating a number of genes involved in controlling and regulating the cell cycle. Mutation in p53 in tumorigenic cells results in uncontrolled cell proliferation and inefficient DNA repair. p53 mutations are estimated to be the most common in tumors of humans, approximately 50% or even greater than that (12).

### Breast Cancer-1 and 2 Genes

Breast cancer-1 and 2 genes are linked to familial breast cancer. Breast Cancer-1 gene consists of 100 Kb DNA and 21 exons. It has a zinc-finger domain like that in the DNA binding proteins. Breast cancer-1 is a tumor suppressor gene. BRCA-2 is located on chromosome 13.

### Tumor Suppressor Genes and their application

Tumor suppressor genes can be studied at the levels of DNA, mRNA, and proteins in the normal and cancerous cells using various methods. Tests for the detection of heterozygosity can be helpful for identifying individuals predisposed to retinoblastoma and other malignancies. Higher frequency of p53 mutations also offers diagnostic and analytical possibilities. PCR amplification can be used to study the changes along with recent methods such as RNase protection assays, single-strand conformational polymorphism or denaturing gel electrophoresis. Immunometric–type assays are quite good at measuring altered p53 in tumor cell line lysates and tissue homogenates (13, 14).

### Molecular pathology: Diagnosis of cancer

One of the primary challenges in the clinical management of cancer patients is to establish the correct diagnosis. As a result, a number of technologies have been developed and are now routinely employed to subtype molecularly cancers. These include immunohistochemistry, immunofluorescence, and the analysis of DNA and RNA extracted from the lesion-through *In situ* hybridization and fluorescent in situ hybridization (FISH). The cancer specimen is then subtyped using different approaches of molecular biology including Sanger sequencing, pyrosequencing, allele-specific PCR. Cancer genotyping is performed by snapshot assay, mass spectroscopy based assays and next generation sequencing (based on fluorescence or semiconductor detectors). The introduction of next-generation sequencing is serving to uncover the true diversity of cancers as well as to define recurring mutations targeted with new therapies. Such genomic-level analyses will continue to have an impact for many years (15, 16).

### Cancer treatment – then & now

Different treatment techniques and therapies have been applied for the treatment of cancer at different times. Some of the most common methods used include surgery, radiation therapy, chemotherapy, hormonal therapy, immunotherapy, adjuvant therapy, targeted-growth signal inhibition, drugs that induce apoptosis, nanotechnology, RNA expression and profiling, and the latest being CRISPR (17). Few of these shall be later discussed in this review.

Cancer cells can also be killed by gene replacements or by knocking out oncogenes. Oncolytic viruses can be used in combination with chemo-therapeutic agents to destroy cancer cells as well (18).

### Retroviral therapy for cancer

Apart from the conventional methods, retroviruses (RVs) have also been used in cancer therapy. RVs can be and have been used for transferring genes to mammalian cells. Most popular RVs are the ones derived from the Moloney Murine leukaemia virus (MoMLV). In the last twenty years, artificial evolution of RVs has enabled their applications in developing transgenic animals, stable delivery of siRNA and clinical trials for gene therapy. The great potential of RVs was discussed in recent reports about the successful clinical trials of gene therapy in patients with severe immunodeficiency disease. However, there are some probable risks inherently related with that (19). A comparative experiment was done by designing two groups of vectors. One was intrinsically replicative, the other was defective and so it had a helper retrovirus with it. Under in vitro conditions, the replicative viruses achieved more than 85% transduction while the other transduced only less than 1%. This experiment clearly indicates the potential of RRVs for developing cancer gene therapy (20).

### Retroviral tagging and insertional oncogenesis

Recently, interest in investigating retroviral vector insertions (insertional oncogenesis) has been growing. For many years, viral insertion sites were used to identify possible oncogenes and cancer signaling pathways. The scope of this approach has been broadened by techniques like insertion site cloning by high throughput PCR, availability of genetically modified animals and with the completion of mouse genome project (21, 22). However, numerous investigators have identified hundreds of common sites. These integration sites are usually associated with cancer genes in MoMLV-induced murine haematopoietic malignancies (23–25). Generally, majority of the insertions exist outside the coding regions. Therefore, only less than 10% of the RISs can be considered as the accepted tumor suppressor genes. Quite interestingly, around 17%–18% of RISs are targeted transcription factors (19).

### Problems with Retroviral Therapy

There are some safety concerns associated with the retroviral gene therapy addressed. Some possible solutions to this problem could be targeted infection, transcriptional targeting, local delivery, co-transduction of retroviral vector with a suicidal gene, specifically targeted retroviral insertion, and SIN vectors. These procedures, with both viral and non-viral systems, can be used in protocols for gene therapy. Nevertheless, insertional oncogenesis remains the major concern regarding retroviral gene therapy (26). A safe and fast means of targeted approach could be the use of foamy viruses (27, 28). These viruses are harmless to humans and yet have a wide range of hosts (29–31). However, for ex vivo cell-based gene therapy, insertional oncogenesis can be avoided by pre-screening of transduced cells in order to select only those cells (clones) which have the transgene only at a desirable site of the chromosome (26).

### Molecular biology techniques for the treatment of cancer

Initially, homologous recombination was used to inactivate the target gene. This was done for characterizing the function of genes. This method was not as productive as it was not efficient in introducing constructs onto the target site. It was very lengthy process, the selection process was laborious and there were many severe mutagenic effects of this method (32).

### RNA Interference

One of the methods used in cancer therapy is RNA interference. RNA interference involves the use of small non-coding RNA that can bind with other mRNAs and can inhibit their translation into proteins. This can result in the loss of function of genes. In cancer therapy, these RNAi can be used to destroy the function of cancer genes that prevent cancer from spreading (33–35). The RNAi method is fast, cheap and it has a high efficiency so it replaced homologous recombination method. Still, it has drawbacks that include incomplete knockdown and temporary prevention of gene function. It also gives off-target effects unpredicted. Cancer recurrence was also seen in some cases. All these problems lead the researchers to explore new methods to alter the gene function (36).

Genome editing is a far better and new technique to treat cancer. Scientists used engineered nucleases that have specific domains that can bind to the target site followed by its cleavage (37, 38). These nucleases were able to induce double-strand breaks (DSBs) in the target followed by the activation of DNA repair mechanisms. Two types of nucleases are used that include programmable nucleases like Zinc Finger Nucleases (ZFNs) and transcription-activator-like effector nucleases (TALENs). These nucleases were successful in genome editing for curing cancer in different animal models (39).

### Zinc finger proteins (ZNFs)

#### ZNFs are the first nucleases to be used for gene editing

These are found as DNA binding domains in eukaryotes. These are made up of 30 amino acids modules arranged in the form of an array of Cys2-His2 DNA-binding zinc fingers. These modules are used to a nuclease domain of FokI (37, 40, 41). The modules consist of 3–6 zinc fingers that can identify nucleotide triplets (42, 43). The FokI nuclease functions only as dimers so a pair of zinc finger nucleases is needed to target any region in the genome. One ZFN will identify the sequence upstream of the genome region to be modified and other will identify downstream sequence (44). These arrays bind to nearby DNA sequences that are in the opposite strands to induce a double-stranded break in the specific region. The breaks are then repaired by different methods that can cause different changes in the specific region like point mutations, indels or translocations. The ZNFs are custom designed so that they recognize all possible nucleotides and any specific region of DNA. (42, 45, 46).

#### Transcription activator-like effector nucleases (TALENs)

TALENs are similar to zinc finger nucleases, as they also need DNA binding motifs and the same nuclease to edit the genome. The difference lies in the recognition of nucleotides as the TALENs domain identifies only one nucleotide instead of a triplet. The interactions between the TALEN domains and their target sites are stronger as compared to ZNFs. It is easier to design TALENs as compared to designing of ZNFs (46, 47). Using TALENs for cancer therapy is very effective method. It needs two specifically engineered TALENs that can identify the sequences of DNA in the target gene on the opposite strands. It dimerizes the FokI nuclease cleavage domain in the TALENs, cleaving the sequence in the target gene (48–50). This causes double-stranded DNA breaks in the targeted gene. The lesion due to the DNA break is repaired by the end-joining DNA repair system. The target gene is altered due to the change in the reading frame. This method can also be used to remove the already present mutations. This gene editing technology can be used to treat the cancer cell lines efficiently as it can target any gene in the genome. Complex cancer genes can be treated by using TALENs (51).

#### CRISPR/CAS9 system: A powerful tool for genome editing in cancer

A powerful genome-editing technology known as Clustered regularly interspaced palindromic sequences-acronym CRISPR, is now eclipsing all other genome-engineering techniques. This revolutionary technique allows researchers to accomplish targeted manipulation in any gene (DNA sequence) in the entire genome of any organism in vitro or now even directly in endogenous genome, thus helping to elucidate the functional organization of genome at systems level and identifying casual genetic variations. CRISPR plays a vital role in detection of cancers (52).

#### Mechanism of CRISPR-Cas9 in cancer treatment

**Fig. 1:**
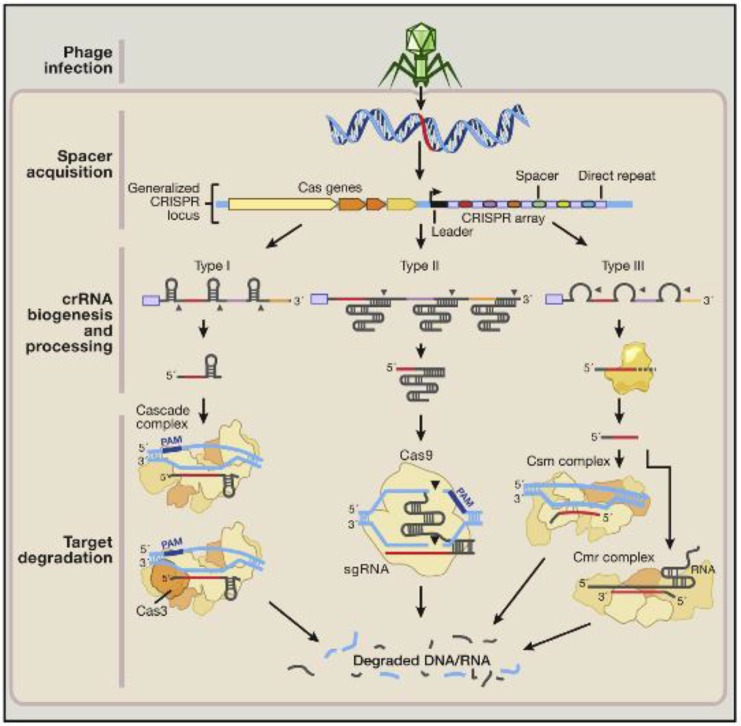
Natural mechanisms of microbial adaptive immune CRISPR Systems: 1. Phage infection, 2. Spacer acquisition, 3. cRNA biogenesis, and processing (52)

If cancer causing gene is known, cancerous cells can be treated with CRISPR-Cas9 system which helps in gene deletion and replacement with a normal gene. Yin et al in his paper discuss the injection process using CRISPR-Cas9 system to cut and introduce gene into liver cells. CRISPRCas9 is more effective for single gene mutation cancers and is mostly delivered in vitro in a particular location. In case of metastatic cancers, in vitro delivery becomes difficult. CRISPR primarily constitutes two biological components: Engineered single guide RNA (sgRNA) and Cas9. A small guide RNA (crRNA and tracrRNA) is used to recognize the complementary sequence-specific target flanked by proto-spacer adjacent motif (PAM) and it guides endonucleases i.e. Cas9 to cleave this sequence (53). Different CRISPR-Cas systems have been grouped majorly into three types (I–III) and subtypes (as I-E) depending on diverse bacterial and archeal repeat sequences, as genes, and their mode of action (54). Type I and III systems share a common mechanism of processing of pre-crRNAs (to crRNA) via specialized Cas endonucleases, and on maturation, each crRNA complex with multi-Cas protein is capable of recognizing and cleaving target sequences which are complementary to crRNA. In contrary to this, Type II system is considered the heart of genome engineering tool because it involves reduced number of Cas enzymes (Fig. 1).

**Fig. 2:**
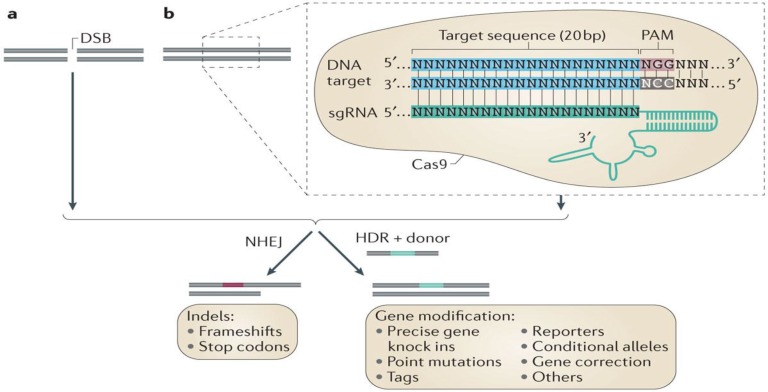
Genome editing via CRISPR-Cas9 system. a) Double-strand breaks in DNA sequence can be repaired by cellular DNA repair mechanisms: the non-homologous end joining (NHEJ) or homology-directed repair (HDR)

It requires non-coding tracrRNA which triggers the processing of pre-crRNA via dsRNA specific Ribonuclease RNase III and cas9 protein (only protein carrying out effective crRNA-mediated silencing of target) (55). CRISPR loci are predominantly composed of multiple repeated sequences (approx. 21–48 bp) interspaced by variable spacer sequences (approx. of 2–72 bp) and Cas genes situated alongside CRISPR locus. First, this CRISPR locus array is transcribed as single RNA, processing of this released pre-RNA (from within repeat sequences) into singular shorter units of CRISPR RNAs (crRNAs) using host proteins is done. Mature crRNA effectively binds to nucleases i.e. Cas proteins, thus this complex of crRNA-cas9 helps in recognizing and then cleaving the target invading DNA or RNA having complementarity to crRNA (sites called protospacers). A motif of 2–5 nucleotide called as PAM is located alongside protospacer in CRISPR-Cas systems I & II (54). PAM, a nucleic acid sequence made up of NGG or NAG trinucleotide for Cas9, flanks at 3’ end of the DNA target site, helps Cas9 in its specific cleavage activity and also facilitates Cas9 in distinguishing self-versus non self-bacterial sequences as PAM (52). SpCas9 (called as *Streptococcus pyogenes* Cas9) is being in use currently for effective genome editing in eukaryotic organisms including humans. PAM located downstream of target is only sequence allowing cas9 target site selection. Cas9 proteins vary in their size and sequence and have common domains as HNH and RuvC endonuclease to cleave two strands of target DNA (Fig. 2).

Cas9HNH domain is specified for cleavage of strand complementary to guide (target) sequence while Cas9 RuvC domains for non-complementary (non-target) strand, In addition, Cas9 holds some conserved arginine-rich sites for binding of nucleic acid (53). Target recognition by this crRNA directs the silencing of foreign sequences by means of case proteins that function in complex with crRNAs (52, 54).

The *Streptococcus* pyogenes-derived CRISPR–Cas9 RNA-guided DNA endonuclease is localized to a specific DNA sequence via a single guide RNA (sgRNA) sequence, which base pairs with a specific target sequence that is adjacent to a protos-pacer adjacent motif (PAM) sequence in the form of NGG or NAG.

On induction of double-stranded breaks or nicks at targeted regions, repairing is done by either *Non-homologous end joining (NHEJ) or Homology-directed repair (HDR) pathway*. NHEJ is an error-prone repair mechanism where joining of broken ends takes place, which generally results in heterogeneous indels (insertions and deletions) whereas HDR is a precise repair method in which homologous donor template DNA is being used in repair DNA damage target site (53).

#### Advantages of CRISPR over traditional methods

The CRISPR/Cas9 system is preferred over the ZNFs and TALENs because of many advantages. Firstly, the target design process is simpler for CRISPR as it depends upon the ribonucleotide complex formation instead of DNA recognition. It can be designed easily and it is much cheaper than designing nucleases as this does not need different proteins for each target and eliminates laborious cloning steps. This can be used to target any specific sequence in the genome. The CRISPR system is much more efficient than ZFNs and TALENs. The RNA encoding Cas protein can be injected directly for modifying the host genome. It is not so lengthy and laborious process as compared to traditional methods (12, 51). By using CRISPR, we can introduce multiplexed mutations. Many genes can be mutated simultaneously by injecting with many gRNAs. This process is faster as compared to other methods. It does not introduce sensitivity to DNA methylation so it can be used if the target site is GC rich.

## Conclusion

Molecular biology has rapidly evolved in the last decade than it has ever before. Different cancer treatment techniques are emerging and succeeding and with the development of ZNFs, TALENs, and CRISPR, scientists are able to target any sequence in the genome, even multiple genes. This will provide immense help in the treatment of diseases like cancer, avoiding the risks caused by the previous methods.

## Ethical considerations

Ethical issues (Including plagiarism, informed consent, misconduct, data fabrication and/or falsification, double publication and/or submission, redundancy, etc.) have been completely observed by the authors.
